# Impressive Words: Linguistic Predictors of Public Approval of the U.S. Congress

**DOI:** 10.3389/fpsyg.2016.00240

**Published:** 2016-02-23

**Authors:** Ari Decter-Frain, Jeremy A. Frimer

**Affiliations:** Department of Psychology, The University of WinnipegWinnipeg, MB, Canada

**Keywords:** impression formation, language, LIWC, the U.S. Congress, agency, communion

## Abstract

What type of language makes the most positive impression within a professional setting? Is competent/agentic language or warm/communal language more effective at eliciting social approval? We examined this basic social cognitive question in a real world context using a “big data” approach—the recent record-low levels of public approval of the U.S. Congress. Using Linguistic Inquiry and Word Count (LIWC), we text analyzed all 123+ million words spoken by members of the U.S. House of Representatives during floor debates between 1996 and 2014 and compared their usage of various classes of words to their public approval ratings over the same time period. We found that neither agentic nor communal language positively predicted public approval. However, this may be because communion combines two disparate social motives (belonging and helping). A follow-up analysis found that the helping form of communion positively predicted public approval, and did so more strongly than did agentic language. Next, we conducted an exploratory analysis, examining which of the 63 standard LIWC categories predict public approval. We found that the public approval of Congress was highest when politicians used tentative language, expressed both positive emotion and anxiety, and used human words, numbers, prepositions, numbers, and avoided conjunctions and the use of second-person pronouns. These results highlight the widespread primacy of warmth over competence as the primary dimensions of social cognition.

## Introduction

What demeanor makes the best impression within a professional setting? Is playing up agentic or communal aspects of personality more effective? Past research has found that agency and communion are the primary dimensions of social cognition (Cuddy et al., 2008): they explain most of the variance in how people judge one another (Rosenberg et al., 1968; Fiske et al., 2002; Cuddy et al., 2008). Communion tends to be the primary dimension of social cognition (Wojciszke et al., 1998). That is, when trying to make a good impression, coming across as warm and kind seems to be more important than coming across as competent and hard working. However, the primacy of communion over agency may be limited to personal contexts, such as among friends and family; in professional settings, where merit and mastery matter more, agency may be more important (Abele and Brack, 2013). The goal of this research is to examine—in a real world, professional context—the roles of agentic and communal language in making a favorable impression.

We define agency as a desire to get ahead and differentiate oneself from others (Bakan, 1966). It is a concern for competence, intelligence, skill, creativity, achievement, power, mastery, and assertiveness. To lack agency is to be weak, submissive, incompetent, and likely to fail. We define communion as a desire to get along and be a part of a larger social or spiritual entity. It is concern for friendliness, helpfulness, sincerity, trustworthiness, togetherness, solidarity, and intimacy. To lack communion is to be hostile, manipulative, and antisocial.

The U.S. Congress has a complex relationship with its electorate. In November 2014, public approval ratings of the U.S. Congress reached an all-time low, with just 9% of Americans expressing satisfaction with their own, elected government. What might be responsible for this shaky relationship between the people and its government? One possible explanation is that Congress has become incompetent, failing to pass legislation that serves the public's interests. However, the available data do not support this notion: the number of bills passed by Congress only weakly—and negatively—predicts its public approval (Ramirez, 2009). A recent article in the Washington Post summarized the mystery surrounding public approval of U.S. Congress: “Even when Congress does things, people still hate it” (Bump, 2015).

What, then, might explain public approval of Congress? Examining trends in public approval over the past few decades, one quickly notices that approval has fluctuated dramatically. As recently as 2001, approval reached 84%, before beginning a precipitous decline toward recent record lows. Societal and global factors, such as the September 11, 2001 attacks, may be responsible for the high approval in 2001. When the country is under threat, the people rally behind the government. However, 9/11 cannot explain why public approval of Congress was relatively high (over 40%) before 2001. What else may be at play?

We suggest that part of the answer lies in the demeanor of politicians, the manner in which the government communicates and the words they choose. A recent study found that politicians' choice of words play a significant role in explaining public approval (Frimer et al., 2015). Specifically, prosocial language of members of U.S. Congress strongly predicted public approval. Language and approval followed the same trajectory over the period under study. Both levels of prosocial language and public approval increased from 1996 to 2001, then began a precipitous decline over the next 7 years, before settling in at low levels since. Americans listen to their government. A surprisingly large number of Americans (47 million, according to a recent poll, Eggerton, 2013), watch televised debates of U.S. Congress on C-SPAN at least once a week. And media tone may also play a role in transmitting the signal from Congress to the public (Frimer et al., 2015). Coming across as warm and prosocial, by using words like *gentle, contribute, trust*, and *cooperate*, may help governments make a favorable impression upon their electorate.

The previous paper (Frimer et al., 2015) examined the role of just one linguistic category (prosocial words) in explaining public approval of Congress, raising questions about what other linguistic categories may be implicated. The present research expands this inquiry by examining the linguistic predictors of public approval of U.S. Congress through both theory-driven and data-driven approaches. Our theory-driven approach seeks to predict approval using the “Big 2” dimensions of social cognition: agency and communion. We follow with an exploratory data-driven approach to understanding impression formation in Congress as a tentative check on whether current social cognitive theory may miss important aspects of impression formation.

To be clear, our approach is correlational, precluding the possibility of causal inference. That is, our data cannot say whether Congressional language influences public approval. The opposite directionality is also possible: rising public approval could change the manner in which politicians communicate. And third variables, such as the effects of special interest groups, global or domestic events, and so on, could influence both politicians' language and their public approval. However, Frimer et al. (2015) found a 6 months' time lag between language and approval, meaning that what Congress says today best predicts their public approval 6 months into the future. Given that causality can only operate forwards in time, this finding may mean that Congressional language can influence the public. Moreover, this effect persists when controlling for (a) societal and global factors, such as unemployment levels and events like 9/11, (b) government competence, operationalized as the number of bills passed, (c) and even bipartisan cooperation. These findings suggest that language may play a causal role. We include the same control variables in our analysis to test whether the effects of language have a direct link to public approval. However, our present goal is to merely examine linguistic correlates of public approval, the presence of which would be consistent with, but would not establish, a causal link. At the same time, the absence of a correlation would be inconsistent with causal claims.

We examined the linguistic predictors of public approval of U.S. Congress using the standard set of linguistic categories established in previous research (Tausczik and Pennebaker, 2010) and built into Linguistic Inquiry and Word Count (LIWC; Pennebaker et al., 2007). We started with a theory-driven approach, then followed with a data-driven approach to check for possible blind spots in extant theory.

Our theory-driven approach examined links between agentic and communal language in Congress and its public approval. Coming across as agentic, communal, or both can make a good impression. On the other hand, appearing agentic but not communal can elicit envy in others, and appearing communal but not agentic can elicit pity (Cuddy et al., 2007). Highly respected people, such as individuals who received awards for their charity, tend to be high in both agency and communion (Frimer et al., 2011). Agency/Communion theory suggests that the government's use of both highly agentic and highly communal language will predict their public approval. *Hypothesis 1* states that levels of both agentic and communal language by members of the U.S. Congress will positively predict public approval.

Agency and communion are meta-constructs, high level abstractions that summarize multiple operationalizable constructs. At such a high level of abstraction, these meta-constructs may not always be fine-grained enough to explain some impression formation processes. For example, an individual may value relationships for self-centered, Machiavellian reasons. This person might appear to be high in communion even though he/she has little interest in improving the welfare of others. Accordingly, following Frimer et al. (2011), we propose that communion has at least two components—a desire to *belong to a social group* and a desire to *benefits others (prosociality)*. Belongingness refers to intimacy with friends, family, and group members, whereas prosociality refers to advancing the interests of the other people. The two tend to go together, but they are distinct motives. And each motive may be differentially implicated in social cognition.

Our bifurcation of communion aligns with Schwartz (1992) distinction between conservation values (security, tradition, and conformity) vs. self-transcendent values (benevolence, universalism), with McAdams' (1993) distinction between narrative themes of love/friendship vs. caring/help, and with Leach et al. (2007) distinction between sociability and morality. When forming impressions of others, people may alter their weightings of information about sociability, morality, and competence depending on the purpose of their evaluation. For example, when deciding whether to trust a stranger with personal information, people may rely primarily on information about morality, whereas people rely on sociability information when deciding whether or not to invite a stranger to a party (Brambilla et al., 2011).

Generally, people may favor a belonging motive in people they know personally, because the self is the object of the desire for intimacy and affiliation. In more socially distant others, the belonging motive may carry less favor, and may even come across as flaky. Some previous research suggests that prosociality is more important than belongingness when trying to make a good impression in distant others: People who won a national award for their charity (a socially distant context) are differentiated from ordinary people in their prosocial motive, but not in terms of their belonging motive (Frimer et al., 2011). Given the remote relationship between the U.S. Congress and the public, these findings suggest that prosocial language will better predict public approval of U.S. Congress than will communal language that includes belongingness (*Hypothesis 2;* Congress and the public, 2015).

Does presenting an agentic or a communal demeanor make a more positive impression? Communion seems to be the primary dimension of social cognition in most settings (Cuddy et al., 2008). Politicians seen as beneficent tend to garner the most support (Cislak and Wojciszke, 2008). This leads to *Hypothesis 3a*: communal language will be a stronger predictor of public approval than will agentic language. However, agency may be more important than communion in professional settings (Abele and Brack, 2013). Given that U.S. Congress is a professional context, one might expect that using agentic language to be more important than using communal language for bolstering public approval. For example, perceptions of candidates' competence (agency), inferred from images of their faces, predict election outcomes (Todorov et al., 2005). The competing *Hypothesis 3b* is that agentic language will be a stronger predictor of public approval than will communal language.

Theory can sometimes have blind spots, failing to recognize the importance of certain concepts. We followed our theory-driven approaches with an exploratory, bottom-up, data-driven approach to understanding public approval of U.S. Congress. To test for these more specific predictors, we conducted an exploratory stepwise regression analysis, in which we entered all 63 LIWC categories.

More generally, *how* might the words of Congress influence public sentiment? Two possibilities come to mind. The first is a direct mechanism: Americans may watch televised debates of Congress on C-SPAN. A surprisingly large number (47 million) of Americans watch C-SPAN at least once a week (Hart Research Associates, 2013). With roughly 80 million Americans voting in midterm (Congressional) elections, the extent of C-SPAN viewership is substantial. The second mechanism is indirect: The media may watch Congressional debates, be influenced by the language used, and resultantly convey Congress in a positive or negative light in editorial columns. Once sentiments about Congress are seeded in the population, they may spread through a process of social contagion. We conclude our analyses by testing whether media portrayal mediates the link between congressional language and public approval.

## Materials and methods

### U.S. congress word corpus

Since 1996, U.S. Congress has transcribed all of the in-session floor debates and made them publicly available from the U.S. Government Publishing Office (Congressional Record, 2015). Using an API, we downloaded transcripts of all the words spoken during floor debates of the U.S. House of Representatives between January 1996 and November 2014, inclusive. We excluded files marked as “extension of remarks,” which are words that were not actually spoken out loud on the floor, but rather entered into the record after the fact. In total, House members uttered 123,781,226 words. Each transcript contained all the words spoken in a single month. To avoid unreliable measurements, we excluded the 22 transcripts that contained fewer than 5000 words. Remaining were 206 transcripts (months), with an average word count of 600,840 (*SD* = 353, 784). Since public officials consent to have their words entered into the public record, we did not ask an ethics committee to review this study.

### Public approval of the U.S. congress

During 198 of the 227 months (87%) between January 1996 and November 2014, Gallup polled the U.S. public on whether they “approve or disapprove of the way Congress is handling its job”). For each survey, Gallup interviews a minimum of 1000 U.S. adults 18 years or older, randomly selected from all 50 US states and the District of Columbia. They weight their results to correct for unequal selection probability, nonresponse bias, and double coverage of landline and cellphone users. They also weight their data according to demographics from the current U.S. census^1^. Following Frimer et al. (2015), we averaged all the polls taken in a given month, and handled missing data with linear interpolation. Public approval averaged 33% (*SD* = 15%). We also note a limitation of this dataset: It is dichotomous. Approval of Congress may be a continuous construct, and we may miss some variability by relying on a dichotomous measure.

### Text analysis

We content-analyzed each transcript using LIWC (Pennebaker et al., 2007). We used the 2007 LIWC dictionary, which has 63 word categories. LIWC simply counts up the number of words in a target transcript that match any of the words in a particular category, and calculates a density score: *density* = *#matches*/ *#words*). Past research demonstrated each category's reliability and validity (see Tausczik and Pennebaker, 2010).

We also used the prosocial words dictionary as an additional word category. It contains 127 words conveying content about collective interests and helping others. Past research developed, introduced, and validated this dictionary (Frimer et al., 2014, 2015).

### Agency and communion coding

We used the prosocial words dictionary to operationalize the prosocial facet of communion. And we used the standard LIWC categories to operationalize the broader agency and communion constructs. The standard LIWC categories do not include agency and communion, *per se*. However, some of the categories imply agentic or communal motives. For example, agency is evident in categories such as *achievement, insight*, and *money*. To operationalize agency and communion, we had five research assistants, each blind to the study hypotheses, code each LIWC category as being agentic and/or communal. (We examined whether our results were robust with respect the measurement of agency and communion and generally found positive evidence. See the Supplemental Materials for details).

Coders first reviewed definitions of agency and communion derived from past research (Fiske et al., 2007; Cuddy et al., 2008). The definition of agency was “a desire to differentiate oneself from others. It is a concern for competence, intelligence, skill, creativity, achievement, power, mastery, and assertiveness. To lack agency is to be weak, submissive, incompetent, and likely to fail.” The definition of communion was “a desire to be a part of a larger social or spiritual entity. It is concern for friendliness, helpfulness, sincerity, trustworthiness, togetherness, solidarity, and intimacy. To lack communion is to be hostile, manipulative, and antisocial.” Coders then reviewed the names of the categories and a short list of representative keywords from each category^2^ (found in Table 1).

**Table 1:** **Agency and communion categories, examples, and coding weights**.

**Category**	**Examples**	**Weight**		**Predicting Public Approval, *r***	**Word Density**	
		**Agency**	**Communion**		**US Congress (%)**	**LIWC 2007 Norms (%)**
1st pers plural	We, us, our	−0.4	1.0	−0.23	1.9	0.7
1st pers singular	I, me, mine	1.0	−0.4	−0.23	1.9	5.0
2nd person	You, your, thou	−0.4	0.8	−0.73	0.3	1.6
3rd pers plural	They, their, they'd	0.0	0.6	−0.19	1.0	0.7
3rd pers singular	She, her, him	0.0	0.8	−0.04	0.6	1.9
Achievement	Earn, hero, win	1.0	0.2	−0.14	2.4	1.6
Adverbs	Very, really, quickly	0.4	0.0	−0.48	2.8	4.8
Affective processes	Happy, cried, abandon	0.0	0.8	−0.08	4.3	5.6
Anger	Hate, kill, annoyed	0.2	−1.0	0.03	0.5	0.6
Anxiety	Worried, fearful, nervous	−1.0	−0.4	0.07	0.2	0.3
Articles	A, an, the	0.0	0.0	0.52	7.6	6.5
Assent	Agree, OK, yes	0.0	1.0	0.01	0.1	1.1
Auxiliary verbs	Am, will, have	0.6	0.2	−0.27	6.9	8.8
Biological processes	Eat, blood, pain	−0.2	0.0	−0.01	1.0	1.9
Body	Cheek, hands, spit	0.2	0.2	0.00	0.2	0.7
Causation	Because, effect, hence	0.4	0.2	−0.42	1.5	1.4
Certainty	Always, never	1.0	0.2	0.21	1.3	1.3
Cognitive processes	Cause, know, ought	1.0	0.4	0.28	14.0	15.0
Common verbs	Walk, went, see	0.2	0.0	−0.40	10.1	15.3
Conjunctions	And, but, whereas	0.0	0.0	−0.33	5.0	5.9
Death	Bury, coffin, kill	0.0	−0.4	0.13	0.2	0.2
Discrepancy	Should, would, could	0.6	0.2	0.00	1.4	1.5
Exclusive	But, without, exclude	0.4	−1.0	0.44	1.7	0.0
Family	Daughter, husband, aunt	−0.2	1.0	−0.12	0.2	0.4
Feel	Feels, touch	0.0	0.8	0.01	0.1	0.6
Fillers	Blah, Imean, youknow	−1.0	0.0	−0.61	0.1	0.4
Friends	Buddy, friend, neighbor	−0.4	1.0	−0.32	0.2	0.2
Future tense	Will, gonna	0.8	0.4	0.29	1.1	1.0
Health	Clinic, flu, pill	−0.2	0.4	0.06	0.6	0.5
Hear	Listen, hearing	0.4	1.0	−0.12	0.8	0.7
Home	Apartment, kitchen, family	0.2	0.8	−0.19	0.6	0.6
Humans	Adult, baby, boy	0.0	0.8	0.61	1.7	0.0
Impersonal pronouns	It, it's, those	0.2	0.0	−0.38	5.4	5.2
Inclusive	And, with, include	0.0	1.0	−0.26	4.9	0.0
Ingestion	Dish, eat, pizza	0.0	0.2	−0.31	0.1	0.5
Inhibition	Block, constrain, stop	−0.2	−0.4	0.16	1.0	0.0
Insight	Think, know, consider	1.0	0.6	0.26	1.5	2.1
Leisure	Cook, chat, movie	−0.4	1.0	−0.25	0.4	1.4
Money	Audit, cash, owe	1.0	0.0	−0.16	2.0	0.7
Motion	Arrive, car, go	0.8	0.2	−0.66	1.8	2.1
Negations	No, not, never	0.0	−0.4	−0.25	1.1	1.7
Negative emotion	Hurt, ugly, nasty	0.0	−1.0	0.00	1.4	1.8
Nonfluencies	Er, hm, umm	−0.8	0.0	−0.05	0.1	0.3
Numbers	Second, thousand	0.2	0.0	−0.15	0.8	2.0
Past tense	Went, ran, had	0.2	0.2	−0.26	2.0	4.1
Perceptual processes	Observing, heard, feeling	0.8	0.8	−0.29	1.3	2.4
Personal pronouns	I, them, her	0.2	0.6	−0.45	5.7	9.8
Positive emotion	Love, nice, sweet	0.0	1.0	−0.14	2.9	3.8
Prepositions	To, with, above	0.0	0.2	0.43	13.4	12.6
Present tense	Is, does, hear	0.6	0.4	−0.42	6.3	8.1
Quantifiers	Few, many, much	0.4	0.2	0.21	2.2	2.5
Relativity	Area, bend, exit, stop	0.2	0.0	−0.52	11.6	13.9
Religion	Altar, church, mosque	−0.4	1.0	−0.18	0.2	0.3
Sadness	Crying, grief, sad	−0.8	0.0	−0.13	0.3	0.4
See	View, saw, seen	0.4	0.4	−0.35	0.3	0.9
Sexual	Horny, love, incest	0.0	1.0	0.08	0.1	0.3
Social processes	Mate, talk, they, child	0.0	1.0	−0.06	8.4	9.4
Space	Down, in, thin	0.2	0.2	−0.31	5.7	6.2
Swear words	Damn, piss, fuck	−0.2	−1.0	−0.06	0.0	0.2
Tentative	Maybe, perhaps, guess	−1.0	0.0	0.32	1.4	2.4
Time	End, until, season	0.6	0.0	−0.42	3.9	5.8
Total pronouns	I, them, itself	0.4	0.4	−0.45	11.1	15.0
Work	Job, majors, xerox	1.0	0.2	0.11	4.1	2.3

*Correlations with approval refer to the association between each category's word density and public approval. Weightings indicate the proportion of coders who judged each category to be indicative of each social dimension*.

For agency, the coders assigned a score of +1 if the category was agentic, −1 if the category implied a lack of agency, 0 if the category was agency-neutral. They did the same, independently, for communion. For example, all five judges assigned a score of +1 on communion for the categories called *family* and *friend*. And all five judges also assigned a −1 agency score to the categories *anxiety* and *tentativeness* in that anxious and tentative people seem to lack agency. And all five judges assigned a score of 0 on both agency and communion to the category *ingestion*.

Coders generally agreed in their judgments (communion ICC = 0.90, agency ICC = 0.82) so we derived weights for agency and communion for each LIWC category by taking the average across the five judgments (see Table 1). Agency and communion weightings were independent, *r* = −0.03, *p* = 0.81. This aligns with the common theoretical understanding of these dimensions as orthogonal (Fiske et al., 2002; Cuddy et al., 2007).

### Third variables

To test whether the link between the language of Congress and the approval of Congress is merely a product of some third variable, we collected data on several factors exogenous to US Congress (e.g., the economy) and several factors endogenous to Congress (e.g., partisan conflict) to act as statistical controls.

#### Exogenous factors

We collected data on several factors exogenous to US Congress that could explain both changes in language and changes in public approval. Among them were the effect of world events, US unemployment, and Americans' expectations about the economy. These are factors that previous research (Ramirez, 2009; Frimer et al., 2015) has examined as possible explanations for public approval of Congress.

##### World events

We reasoned that if world events (e.g., 9/11) were responsible for changes in Congressional rhetoric and in public approval, they also would have had a similar effect on the language of the US President. We estimated the effect of world events by assessing the levels of prosocial, communal, and agentic language in the rhetoric of the President. Between 1996 and 2014, The President held 411 news conferences. We downloaded all conference transcripts for a total of 2,205,168 words^3^. We then analyzed each transcript for prosocial word density using the prosocial words dictionary, then averaged the scores of transcripts in each month (*M* = 1.60%, *SD* = 0.37%). We used the agency and communion coding described in section 1.4 to measure the communal and agentic content of Presidential rhetoric (agency *M* = 0, *SD* = 4.54, communion *M* = 0, *SD* = 5.00).

##### Unemployment

We downloaded employment statistics for persons above the age of 16 from the US Bureau of Labor Statistics (ID LNS 14000000)^4^. Between 1996 and 2014, the US unemployment rate averaged 6.0% (*SD* = 1.8%).

##### Economic expectations

In accordance with past research (Ramirez, 2009), we operationalized public expectations about the economy as the University of Michigan Index of Consumer Sentiment^5^ (ICS). ICS polls are released monthly. It aggregates five measures of consumer confidence; whether (1) they are better off financially than they were 1 year ago, (2) they expect to be better off financially 1 year into the future, (3) they expect business to improve in the coming year, (4) they expect the country's financial situation to improve over the next 5 years, and (5) the present is a good time to buy major household appliances. Over the period of study, scores on this measure ranged from 55.3 to 112.0 (*M* = 86.67, *SD* = 13.99).

#### Endogenous factors

As additional third variables, we also collected data on several factors endogenous to US Congress, such as the composition and functioning of Congress. Among these were the amount of conflict between the parties, the efficacy of Congress, and the demographic composition of Congress.

##### Partisan conflict in congress

Following Ramirez (2009), we defined a partisan vote as one in which at least 75% of Republicans voted one way and 75% of Democrats voted the other. The House voted on 12,563 bills between 1996 and 2014 for an average of 55 votes per month (*SD* = 42). We downloaded and retained data from months that had five or more votes^6^ (200 out of 227 mo, 88% retention). Finally, we operationalized partisan conflict as the proportion of partisan votes per month (*M* = 43%, *SD* = 18%).

##### Congressional efficacy

We operationalized Congressional efficacy as the number of bills passed in a given month. We downloaded summaries of every vote in US Congress^5^. We operationalized bills passed in the House as the number of bills that received the majority of votes. On average, the House passed 38 bills (*SD* = 27) per month.

##### Presidential vetoes

We collected veto counts from the U.S. Senate website. Presidential vetoes occurred infrequently over the period of study (1996–2014 total = 39, *M* = 0.17, *SD* = 0.50)^7^.

##### Congress composition

We collected data on the composition of Congress, both in terms of party and gender. We retrieved the number of female members of Congress each term (*M* = 15%, *SD* = 2%) and the number of Democratic members of Congress each term (*M* = 49%, *SD* = 4%)^8^.

### Mechanism

To examine how Congressional language could influence public opinion, we tested one possible communication channel: media coverage on Congress. We used Frimer et al.'s (2015) measure of Congressional media coverage, defined as the quantity × the valence of news editorials covering Congress in a given month. The quantity measure was defined as the number of news editorials published on Congress available in the Dow Jones & Company's Factiva database. Frimer et al. (2015) then sorted the results by relevance and downloaded the most relevant article for tone assessment. Tone was defined as the (human-coded) valence toward Congress on a nine-point scale, anchored to −4 (*extremely negative*) to +4 (*extremely positive*).

## Results

### Theory-driven approach

**Figure 1:**
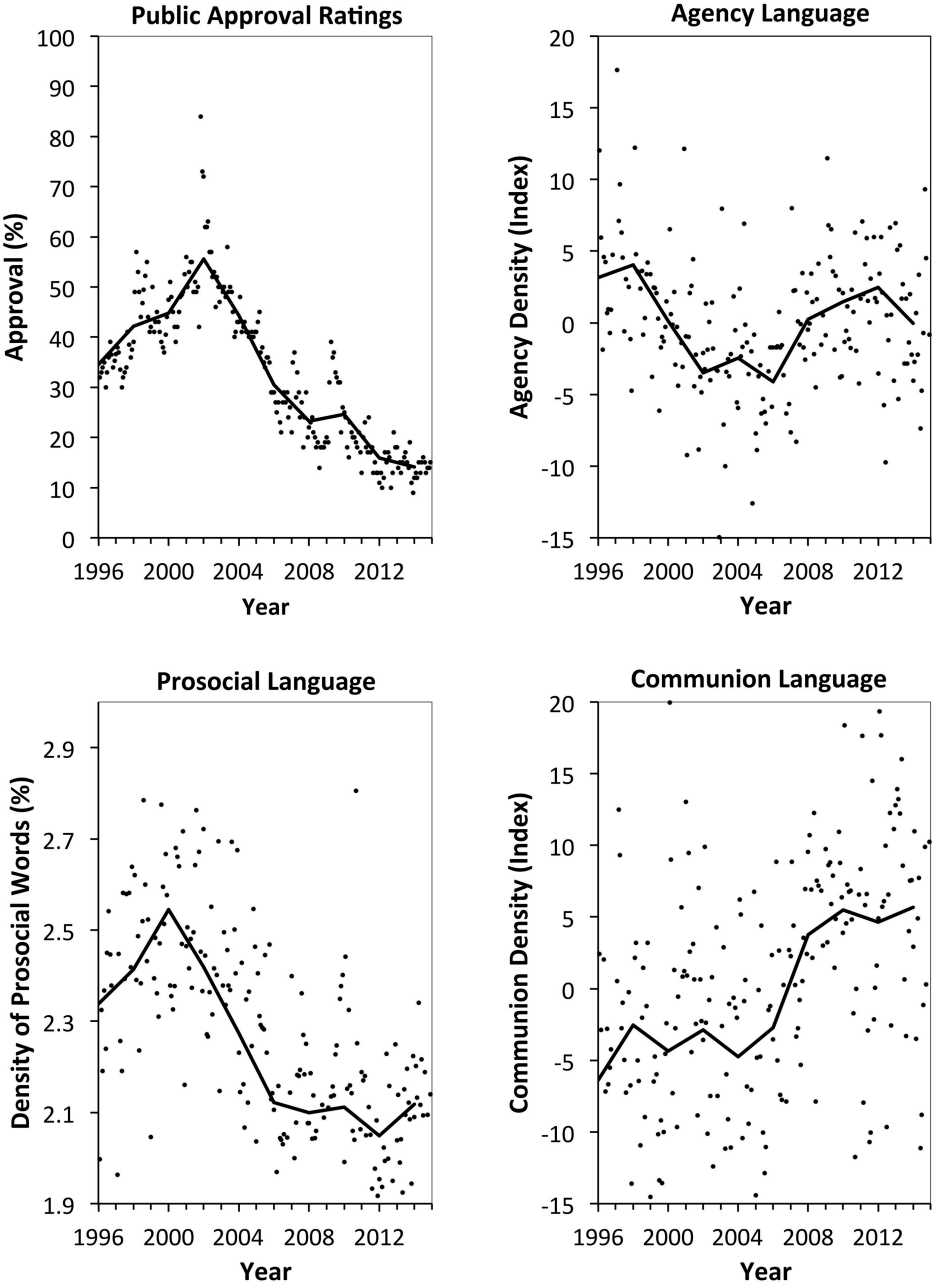
**Language in the U.S. House of Representatives and public approval of Congress over time**. Dots represent scores from individual months. Lines connect 2-year session averages. “public approval” and “prosocial words” taken from Frimer et al. (2015).

After standardizing the word densities (z-scores) from each word category, we calculated agency and communion indexes by multiplying each category's word density by its weight, and summing across all categories. The resulting communion scores ranged from −46.1 to 22.0 (*M* = 0, *SD* = 9.2). Agency scores ranged from −19.8 to 22.0 (*M* = 0, *SD* = 5.1). We then tested whether agentic and communal language in the U.S. House of Representatives positively predicts public approval. Figure 1 shows the trajectory of these types of language and public approval over time. Public approval climbed from 1996 to 2001, declined from 2001 to 2008, then remained low. Prosocial language followed the same trajectory. Interestingly, communal language—based on the broader notion of communion as including a sense of belonging—followed the *opposite* trend to some degree. Communal language was low until 2006 then climbed and remained steadily high after 2008. Communal and prosocial language were negatively related, *r*_(206)_ = −0.14, *p* = 0.04. And agentic language seemed to take a small dip from 2001 to 2006 and otherwise remain high.

We tested the two theory-driven hypotheses in separate regression analyses. *Hypothesis 1* stated that politicians' use of agentic and communal language (broadly defined) would positively predict public approval of the U.S. Congress. First, we examined simple correlations and found that the use of agentic and communal language *negatively* predicted public approval (see Table 2). Entering agency and communion into a regression equation predicting public approval (model 1), we found that only communion predicted public approval—but negatively. (Entering an agency × communion interaction term did not change the result; see model 2). Next, we controlled for third variables. In model 3, we re-ran the model but now including all exogenous and endogenous factors. We found that agency became a significant negative predictor of approval, whereas the effect of communion on public approval ceased to be significant. These results did not support *Hypothesis 1*.

**Table 2:** **Predictors of public approval**.

**Predictors**	**Correlation *r*_(206)_**	**Regression analyses**, **βs**		
		**Model 1**	**Model 2**	**Model 3**
Agency	−0.16^*^	−0.04	−0.04	−0.11^*^
Communion	−0.36^***^	−0.34^***^	−0.35^***^	+0.04
Interaction			−0.04	+0.06
**CONTROL VARIABLES**				
Exogenous Factors				
World Events				
President's Agency				−0.11^*^
President's Communion				−0.04
Unemployment				−0.08
Economic Expectations				+0.08
Endogenous Factors				
Partisan Conflict in Congress				−0.09
Congressional Efficacy				−0.04
President vetoes				−0.12^*^
Congressional Composition				
Party				+0.16^**^
Gender				−0.68^***^
*R*^2^ change		0.13^***^	0.00	0.53^***^

*Results from a test of Hypothesis 1. Politicians' use of communal language negatively predicts approval until controlling for third variables*.

* *p < 0.05*,

** *p < 0.01*,

*** *p < 0.001*.

*Hypothesis 2* states that the prosocial facet of communion will predict public approval whereas communion writ large will less so. To test this, we ran the same basic analyses, only replacing the communion index with the narrower prosocial language category. Table 3 presents the results. Supporting *Hypothesis 2*, we found that prosocial language positively predicted public approval. Agency and the interaction term did not (models 1, 2). Next, we controlled for third, and found that prosocial word density continued to predict public approval (model 3), suggesting that Congress' use of prosocial language remains a robust, independent positive predictor of public approval, whereas communion does not. This evidence supports *Hypothesis 2*.

**Table 3:** **Results from a test of Hypothesis 2**.

**Predictors**	**Correlation *r*_(206)_**	**Regression analyses**, **βs**		
		**Model 1**	**Model 2**	**Model 3**
Agency	−0.16^*^	+0.02	−0.02	−0.06
Prosocial	+0.55^***^	+0.56^***^	+0.57^***^	+0.14^*^
Interaction			+0.08	−0.05
**CONTROL VARIABLES**				
Exogenous Factors				
World Events				
President agency				−0.12^*^
President's prosocial				+0.01
Unemployment				−0.07
Economic expectations				+0.06
Endogenous Factors				
Partisan conflict in congress				−0.11^*^
Congressional efficacy				−0.06
President vetoes				−0.12^*^
Congressional Composition				
Party				+0.15^**^
Gender				−0.59^***^
*R*^2^ change		0.31^***^	0.01	0.36^***^

*Politicians' use of prosocial language positively predicts their public approval, even when controlling for third variables*.

* *p < 0.05*,

** *p < 0.01*,

*** *p < 0.001*.

*Hypothesis 3* made competing predictions about whether agentic or communal language would be the stronger positive predictor of public approval within the U.S. Congress. Comparing the association between communal language and public approval (*r* = −0.36) vs. the association between agentic language and public approval (*r* = −0.16), we found a significant difference, *z* = 2.18, *p* = 0.03. Noting that both associations are negative complicates the interpretation of these results with respect to *Hypothesis 3*. However, when comparing the association between prosocial language and public approval (*r* = +0.55) vs. the association between agentic language and public approval (*r* = −0.16), we found a significant and meaningful difference, *z* = 7.89, *p* < 0.001, lending support to *Hypothesis 3b*. Even in this professional context, prosocial language was a more important predictor of social approval than was agentic language.

Next, we investigated how Congressional language may influence public sentiment—through the media. Using Hayes' bootstrapping procedure (Hayes, 2013), we tested whether media portrayal mediates the effects of Congressional language (prosocial, communal, and agentic) on public approval (see Table 4). Media portrayal explained the link between each of the three linguistic predictors and public sentiment.

**Table 4:** **Direct effects (C-SPAN) and indirect effects (media valence) of linguistic categories on public approval from bootstrapped mediation analyses**.

**Linguistic Category**	**M**	***SD***	**Direct effect**		**Indirect effect**	
			**B**	**95% CI**	**B**	**95% CI**
Agency	0.00	9.15	−0.55	[−0.98, −0.12]	−0.11	[−0.30, −0.02]
Communion	0.00	5.09	−0.54	[−0.79, −0.30]	−0.06	[−0.16, −0.01]
Prosocial	2.26	0.27	+36.30	[29.05, 43.55]	1.82	[0.17, 5.26]

*All three word categories exhibit direct and indirect effects on public approval*.

In a follow-up moderation analysis, we examined whether the effect of agentic and communal language on public approval depends on the demographic make-up of Congress (both in terms of political leaning and gender). In particular, we tested whether the public approved of stereotype-confirming behavior (e.g., communal females, agentic males) or stereotype-disconfirming behavior (e.g., agentic females, communal males). The results from our analyses were mixed, but more so supported the stereotype-disconfirming hypothesis. Our analyses suggested that agency becomes a negative predictor of public approval only when Congress is male-dominated, whereas agency does not predict public approval when Congress was less dominated by males. We also found that communal language garners approval when Congress is dominated by males, but communal language elicits disapproval when it was less dominated by males. Prosocial language consistently predicted higher approval, and the effect was stronger when Congress had fewer males and more Republicans. See the Supplemental Materials for details.

### Data-driven approach

To explore whether extant theory may have missed certain features of impression formation, we conducted a stepwise regression analysis with all 63 LIWC categories as possible predictors of public approval. To limit the potential for false positive results, we set conservative limits of *p* < 0.005 for inclusion and *p* > 0.01 for exclusion in the model. Eight of 63 categories entered the model (see Table 5). Public approval of the U.S. Congress was highest when politicians used tentative language, talked about humans, expressed both positive emotion and anxiety, used prepositions and numbers, and avoided the use of conjunctions and the second person. We interpret these results in the Discussion.

**Table 5:** **Linguistic predictors of public approval of U.S. Congress**.

**Linguistic category**	**Examples**	**Standardized coefficient, β**	***P***
**POSITIVE PREDICTORS**			
Tentativeness	Maybe, perhaps	0.46	< 0.001
Prepositions	To, with, about	0.41	< 0.001
Humans	Adult, baby, boy	0.25	< 0.001
Anxiety	Worried, fearful, nervous	0.24	< 0.001
Positive emotion	Love, nice, sweet	0.24	< 0.001
Numbers	Second, thousand	0.15	0.001
**NEGATIVE PREDICTORS**			
2nd person	You, your, thou	−0.18	0.005
Conjunctions	And, but, whereas	−0.26	< 0.001

*Results from an exploratory analysis*.

To test whether each of the categories predicting approval did so directly or indirectly through media portrayal, we ran mediation analyses for each of the eight significant predictors, testing media portrayal as a mediator (see Table 6). The results suggest that media portrayal helps explain how the use of prepositions, conjunctions, and human words could influence the public's feelings toward Congress.

**Table 6:** **Direct effects (C-SPAN) and indirect effects (media valence) of linguistic categories on public approval from bootstrapped mediation analyses**.

**Predictors of approval**	**M**	***SD***	**Direct effect (C-SPAN)**		**Indirect effect (media)**	
			**B**	**95% CI**	**B**	**95% CI**
**POSITIVE PREDICTORS**						
Tentativeness	1.68	0.14	**+33.72**	**[17.88, 49.56]**	+2.22	[−0.70, 8.53]
Prepositions	14.69	0.26	**+29.27**	**[22.34, 36.20]**	**+1.90**	**[0.30, 5.02]**
Humans	1.37	0.24	**+29.81**	**[21.14, 38.48]**	**+2.40**	**[0.45, 6.03]**
Anxiety	0.20	0.05	**+81.34**	**[36.92, 125.75]**	−2.14	[−17.14, 12.42]
Positive emotion	3.04	0.19	−4.51	[−16.40, 7.38]	+0.15	[−4.17, 3.11]
Numbers	0.86	0.08	−17.22	[−45.31, 10.88]	−0.11	[−9.68, 6.41]
**NEGATIVE PREDICTORS**						
2nd person	0.33	0.11	−**83.05**	**[**−**99.31**, −**66.78]**	−3.98	[−11.56, 0.14]
Conjunctions	5.37	0.17	−**43.19**	**[**−**55.34**, −**31.03]**	−**3.51**	**[**−**8.77**, −**0.45]**

*Bolded numbers are significant*.

## Discussion

What demeanor makes the best impression? We examined whether the usage of different categories of words predict social approval in a pressing real world setting—the U.S. Congress. Over the time in which transcripts of Congress were available (since 1996), public approval has varied dramatically. Using both theory-driven and theory-driven approaches, we found that several linguistic categories were predictive of public approval. For example, public approval is highest when public officials talk helping and humans, express positive emotion, and use tentative language.

### Theory-driven approach

The theory-driven component of this paper examined two hypotheses concerning the linguistic predictors of public approval of Congress. *Hypothesis 1* arose from the theory that admired groups are highly agentic and highly communal (Fiske et al., 2002). It states that agentic and communal language should independently and positively predict approval. Our analyses did not support *Hypothesis 1*.

Noting that communion comes in more than one form, we derived *Hypothesis 2*, which states that only the prosocial, helping component of communion would positively predict approval, whereas the belonging motive would not. Our results supported *Hypothesis 2*. The American public approved of Congress more when its members spoke about helping, but not when they spoke about belonging. When hearing distant others speak, like members of U.S. Congress, observers might value helping more than belonging, because helping would more directly serve the public's interests. These findings highlight the importance of a distinction between belonging and helping as components of communion.

*Hypothesis 3* examined two competing predictions about whether agentic or communal language would be the stronger, more positive predictor of public approval. When examining the differential predictive power of prosocial language and public approval, we found support for *Hypothesis 3b*: even in this professional setting, prosocial language was more positively associated with public approval than was agentic language. This finding is revealing of the far-reaching primacy of the desire for warmth in others over the desire for competence, even in some professional contexts. We also tested whether language exerted a direct effect on public approval, or an indirect effect via media portrayal. We found that communal, agentic, and prosocial language have direct and indirect effects on approval. That is, Congress' words could influence the public through the media and also through other channels, like public viewership of C-SPAN.

We acknowledge that our theory-driven approach carries a number of limitations. For one, our approach is correlational and is therefore subject the third variable and directionality problems. We did control for the effects of several exogenous factors, such as economic conditions, and endogenous factors, such as congressional performance, and still found that prosocial language predicted public approval. We did not, however, examine whether Congressional language still predicts public approval when controlling for the effects of other theoretically relevant demographic variables, such as political orientation, gender, or age of the members of the public. Moreover, unidentified variables may have suppressed a real and meaningful relationship between agentic word use and public approval.

Along the same lines, our approach leaves uncertainty about directionality. For example, the negative relationship between communion and public approval could result from the influence of negative approval ratings on bonding among members of congress. Perhaps as members of Congress feel more under threat from the public, they begin to speak more about Congress itself as a community to which they belong.

Another limitation of this study is that, because we did not predict a negative relationship between communal language and public approval, we did not develop a measure of “belonging” language. Hence, we are unable to confirm our explanation that “belonging” words in the context of Congress appear sappy or flaky. In the Supplemental Materials, however, we did find some additional evidence for this explanation using Hart et al. (2011) agency and communion dictionaries. When we entered the effect of (Hart's) communion on public approval controlling for prosocial words (theoretically leaving only the variability due to “belonging”), we found that it uniquely and *negatively* predicted approval, meaning that the belonging form of communion could elicit disapproval in Congress. Future research could more directly identify belonging and helping as components of communion by developing a “belonging” dictionary to accompany the existing prosocial word dictionary.

### Data-driven approach

In an exploratory analysis, we entered 63 linguistic predictors of public approval, and found that eight predicted public approval in a regression analysis. Each result is merely suggestive until replicated. However, we offer speculative theoretical explanations for these predictors before concluding.

#### Tentativeness

The standard word category that most strongly predicted public approval was tentativeness; the more tentatively Congress spoke, the more the public approved of them (*r* = 0.46). The tentativeness dictionary contains words like “perhaps,” “maybe,” and “guess,” which may indicate a lack of conviction or certainty in the speaker. Consistent with this interpretation, our coding weights suggest that tentativeness may signal a lack of agency (agency weight = −1, communion weight = 0). Alternatively, expressing opinions with uncertainty may exude humility and receptiveness to others' opinions. Tentative expression may disarm an audience, and thus facilitate communication and improve impression formation. In his autobiography, Benjamin Franklin described using tentative language to positive effect when communicating with both colleagues and adversaries:

I grew very artful and expert in drawing people, even of superior knowledge, into concessions, the consequences of which they did not foresee, entangling them in difficulties out of which they could not extricate themselves, and so obtaining victories that neither myself nor my cause always deserved. I continu'd this method some few years, but gradually left it, retaining only the habit of expressing myself in terms of modest diffidence; never using, when I advanced anything that may possibly be disputed, the words *certainly, undoubtedly*, or any others that give the air of positiveness to an opinion; but rather say, *I conceive* or *apprehend a thing to be so and so*; *it appears to me*…This habit, I believe, has been of great advantage to me when I have had occasion to inculcate my opinions, and persuade men into measures that I have been from time to time engag'd in promoting (Franklin, 2006, Ch. 2).

#### Anxiety

Anxious language also positively predicted public approval. Our coders rated anxiety as a strong signal of lacking agency (weight = −1), and as a weaker signal of lacking communion (weight = −0.4). Thus, anxiety may predict public approval because it indicates a lack of agency and communion. Another possible explanation is that expressing anxiety signals threat. When people feel threatened, they tend to accept their position in the social hierarchy (Jost et al., 2004) and seek protection from leaders (Klapp, 1948; Atran and Norenzayan, 2004). Whether members of Congress use anxiety to deliberatively incite fear, or are merely reacting to threatening circumstances remains unclear from the present data.

#### Second-person

We also found that the use of second-person words predicts lower public approval. When Congress used the word “you,” the public tended to like them less. Second person was rated as indicating the presence of communion (weight = 0.8) and the absence of agency (weight = −0.4). Thus, second person may convey the “flakiness” we suggest the public perceives when Congress talks about belonging. Or perhaps the public perceives the use of second-person speech as an accusatory tone, or as an attempt to avoid taking personal responsibility.

#### Prepositions

The use of prepositions also robustly predicted public approval. A Congress that used more words like “to,” “with,” and “above” had higher public approval than one that used these words less. Our coding weightings suggest preposition usage is mostly unrelated to agency (weight = 0) and communion (weight = 0.2). This variable may constitute a theoretical blind spot, the importance of which has gone unidentified by the agency and communion constructs. Psychological correlates of conjunction use include education level and concern with precision (Hartley et al., 2003). Hence, this correlation may, like tentativeness, arise because of an implicit preference among Americans for an educated, precise-sounding government.

#### Humans

When delegates speak about boys, girls, adults, and children, Americans show approval. Our coders thought talking about humans indicated communion (weight = 0.8) but not agency (weight = 0). Human words correlated with prosocial words, *r*_(206)_ = 0.519, *p* < 0.001, suggesting that their usage may indicate the “helping” component of communion. The “humans” LIWC category has be largely unexamined among scholars to date (Tausczik and Pennebaker, 2010). However, one study found that mindfulness-based meditation can increase people's tendency to write about humans, perhaps because meditation increases empathy (Block-Lerner et al., 2007). Speaking about human beings may make politicians come across as empathic.

#### Positive emotion

When people use words like “love,” “nice,” and “sweet,” the public approves. Positive emotion was coded as a strong indicator of communion (weight = 1), but not agency (weight = 0). Again, this suggests that specific components of communion may better predict public approval than the meta-construct as whole. Emotional contagion may also explain this effect. When leaders use positive emotion words, followers' moods improve (Bono and Ilies, 2006). So delegates' words may improve the affect of viewers, which reflects through their approval of Congress.

#### Numbers

Number words positively predict public approval. We are unaware of previous research on the psychological correlates of speaking about numbers, and they also did not receive significant weight on agency (weight = 0.2) or communion (weight = 0). Speculatively, number words may be a symptom of representatives describing *specific* issues and plans. Specifics may imply agentic action. Given the negative or null relationships between agentic language and public approval, these results raise the possibility that some sub-component or sub-form of agency may have a positive effect on impression formation. Perhaps audiences process agency implicitly—an agentic *mode* (e.g., being specific) may make a good impression whereas an agentic *message* (e.g., talking about work) may have less of an effect.

#### Conjunctions

Public approval drops when delegates use more conjunctions; words like “and,” “but,” and “whereas.” Coders did not rate conjunctions as indicative of agency or communion (both weights = 0). This category may represent another blind spot, not picked up by more broad theoretical constructs. This LIWC category remains largely unexplored and so has no known psychological correlates (Tausczik and Pennebaker, 2010). Conjunctions connect two separate phrases in a single, complex sentence. Perhaps people dislike conjunctions because they prefer straightforward communication. If the use of conjunctions were an indicator of complex language, we might expect to find a positive relationship between conjunction word usage and other indicators of rhetorical complexity, such as sentence length (words per sentence) and the density of words with six or more letters. Indeed we found that the density of conjunctions correlates positively with sentence length, *r*_(215)_ = 0.24, *p* < 0.001, but (unexpectedly) correlates negatively with the density of six letter words, *r*_(215)_ = −0.38, *p* < 0.001. While the negative link between conjunction usage and public approval could mean that the public dislikes obfuscating language, the present results are inconclusive on this matter. Future research should examine how conjunction use modifies impression formation and the effect of conjunction use on the social desirability of a speaker.

## Conclusion

What should a person say to make a favorable impression in a professional context? This work examined the linguistic predictors of public approval of U.S. Congress. We emphasize that our findings should be interpreted tentatively, given that we can make no causal inferences from this correlational analysis. Nonetheless, our results suggest that speaking about helping, speaking tentatively, and avoiding the usage of second-person words may help Congress improve its relationship with the population is serves.

## Author contributions

JF developed the theoretical framework of the paper and guided the statistical analysis. JF proposed using coders to create agency and communion scores. JF contributed to writing through substantial editing of each draft of the paper. AD conducted the statistical analysis and designed and carried out the coding task. AD also reviewed theoretically-relevant literature and wrote several drafts of each section of the paper. AD also organized and wrote the references. But authors agree to be accountable for all aspects of the work.

## Funding

This research was supported by a grant from the Social Sciences and Humanities Research Council of Canada to JF (grant number 435-2013-0589).

### Conflict of interest statement

The authors declare that the research was conducted in the absence of any commercial or financial relationships that could be construed as a potential conflict of interest.

## Footnotes

^1^For more information on Gallup polling methods, see http://www.gallup.com/185477/gallup-poll-social-series-work.aspx?utm_source=METHODOLOGY&utm_medium=topic&utm_campaign=tiles.

^2^For category names and keywords, see http://liwc.net/descriptiontable1.php.

^3^Retrievable from www.presidency.ucsb.edu.

^4^Retrievable from www.bls.gov/data.

^5^Retrievable from http://www.sca.isr.umich.edu.

^6^Retrievable from voteview.com/partycount.htm.

^7^Retrievable from www.senate.gov/reference/Legislation/Vetoes/vetoCounts.htm.

^8^Retrievable from www.fec.gov.

## References

[B1] Abele A. E. Brack S. (2013). Preference for other persons' traits is dependent on the kind of social relationship. Soc. Psychol. 44, 86–94. 10.1027/1864-9335/a000138

[B2] Atran S. Norenzayan A. (2004). Religion's evolutionary landscape: counterintuition, commitment, compassion, communion. Behav. Brain Sci. 27, 713–730. 10.1017/S0140525X04000172 16035401

[B3] Bakan D. (1966). The Duality of Human Existence: An Essay on Psychology and Religion. Oxford, England: Rand Mcnally.

[B4] Block-Lerner J. Adair C. Plumb J. C. Rhatigan D. L. Orsillo S. M. (2007). The case for mindfulness-based approaches in the cultivation of empathy: does nonjudgmental, present-moment awareness increase capacity for perspective-taking and empathic concern? J. Marital Fam. Ther. 33, 501–516. 10.1111/j.1752-0606.2007.00034.x 17935532

[B5] Bono J. E. Ilies R. (2006). Charisma, positive emotions and mood contagion. Leadersh. Q. 17, 317–334. 10.1016/j.leaqua.2006.04.008

[B6] Brambilla M. Rusconi P. Sacchi S. Cherubini P. (2011). Looking for honesty: the primary role of morality (vs. sociability and competence) in information gathering. Eur. J. Soc. Psychol. 41, 135–143. 10.1002/ejsp.744

[B7] Bump P. (2015, May 13). Even when Congress does things, people still hate it. The Washington Post. Retrieved from: http://www.washingtonpost.com

[B8] Cislak A. Wojciszke B. (2008). Agency and communion are inferred from actions serving interests of self or others. Eur. J. Soc. Psychol. 38, 1103–1110. 10.1002/ejsp.55426884508

[B9] Congressional Record (2015). Data from: Congressional Record. U.S. and Government Publishing Office. Available online at: http://www.gpo.gov/fdsys/browse/collection.action?collectionCode=CREC

[B10] Congress the public (2015). Data from: Congress and the Public. Gallup. Available online at: www.gallup.com/poll/1600/congress-public.asp.

[B11] Cuddy A. J. Fiske S. T. Glick P. (2007). The BIAS map: behaviors from intergroup affect and stereotypes. J. Pers. Soc. Psychol. 92:631. 10.1037/0022-3514.92.4.631 17469949

[B12] Cuddy A. J. Fiske S. T. Glick P. (2008). Warmth and competence as universal dimensions of social perception: the stereotype content model and the BIAS map. Adv. Exp. Soc. Psychol. 40, 61–149. 10.1016/S0065-2601(07)00002-0

[B13] Eggerton J. (2013). Exclusive: C-SPAN Study Finds Almost Quarter of Cable/Satellite Subs Watch Weekly. Retrieved from: http://www.broadcastingcable.com

[B14] Fiske S. T. Cuddy A. J. Glick P. (2007). Universal dimensions of social cognition: warmth and competence. Trends Cogn. Sci. (Regul. Ed). 11, 77–83. 10.1016/j.tics.2006.11.005 17188552

[B15] Fiske S. T. Cuddy A. J. Glick P. Xu J. (2002). A model of (often mixed) stereotype content: competence and warmth respectively follow from perceived status and competition. J. Pers. Soc. Psychol. 82:878. 10.1037/0022-3514.82.6.878 12051578

[B16] Franklin B. (2006). The Autobiography of Benjamin Franklin (Vol. 1). Retrieved from: http://www.gutenberg.org/files/20203/20203-h/20203-h.htm

[B17] Frimer J. A. Aquino K. Gebauer J. E. Zhu L. L. Oakes H. (2015). A decline in prosocial language helps explain public disapproval of the US Congress. Proc. Natl. Acad. Sci. U.S.A. 112, 6591–6594. 10.1073/pnas.1500355112 25964358 PMC4450383

[B18] Frimer J. A. Schaefer N. K. Oakes H. (2014). Moral actor, selfish agent. J. Pers. Soc. Psychol. 106, 790–802. 10.1037/a0036040 24749822

[B19] Frimer J. A. Walker L. J. Dunlop W. L. Lee B. H. Riches A. (2011). The integration of agency and communion in moral personality: evidence of enlightened self-interest. J. Pers. Soc. Psychol. 101, 149. 10.1037/a0023780 21574724

[B20] Hart C. M. Sedikides C. Wildschut T. Arndt J. Routledge C. Vingerhoets A. J. (2011). Nostalgic recollections of high and low narcissists. J. Res. Pers. 45, 238–242. 10.1016/j.jrp.2011.01.002

[B21] Hartley J. Pennebaker J. W. Fox C. (2003). Abstracts, introductions and discussions: how far do they differ in style?. Scientometrics 57, 389–398. 10.1023/A:1025008802657

[B22] Hayes A. F. (2013). Introduction to Mediation, Moderation, and Conditional Process Analysis: A Regression-Based Approach. New York, NY: Guilford Press.

[B23] Jost J. T. Banaji M. R. Nosek B. A. (2004). A decade of system justification theory: Accumulated evidence of conscious and unconscious bolstering of the status quo. Polit. Psychol. 25, 881–919. 10.1111/j.1467-9221.2004.00402.x

[B24] Klapp O. E. (1948). The creation of popular heroes. Am. J. Sociol. 54, 135–141. 10.1086/220292

[B25] Leach C. W. Ellemers N. Barreto M. (2007). Group virtue: the importance of morality (vs. competence and sociability) in the positive evaluation of in-groups. J. Pers.Soc. Psychol. 93, 234–249. 10.1037/0022-3514.93.2.234 17645397

[B26] McAdams D. P. (1993). The Stories We Live By: Personal Myths and the Making of the Self. New York, NY: Guilford Press.

[B27] Pennebaker J. W. Booth R. J. Francis M. E. (2007). Linguistic Inquiry and Word Count: LIWC [Computer software]. Austin, TX: LIWC.net.

[B28] Ramirez M. D. (2009). The dynamics of partisan conflict on congressional approval. Am. J. Pol. Sci. 53, 681–694. 10.1111/j.1540-5907.2009.00394.x

[B29] Hart Research Associates (2013). C-SPAN at 34:a Bi-Partisan, Politically Active Audience that Continues to Grow. Available online at: http:/series.c-span.org/About/The-Company/Press-Releases/

[B30] Rosenberg S. Nelson C. Vivekananthan P. S. (1968). A multidimensional approach to the structure of personality impressions. J. Pers. Soc. Psychol. 9, 283. 10.1037/h0026086 5670821

[B31] Schwartz S. H. (1992). Universals in the content and structure of values: theoretical advances and empirical tests in 20 countries. Adv. Exp. Soc. Psychol. 25, 1–65. 10.1016/S0065-2601(08)60281-6

[B32] Tausczik Y. R. Pennebaker J. W. (2010). The psychological meaning of words: LIWC and computerized text analysis methods. J. Lang. Soc. Psychol. 29, 24–54. 10.1177/0261927X09351676

[B33] Todorov A. Mandisodza A. N. Goren A. Hall C. C. (2005). Inferences of competence from faces predict election outcomes. Science 308, 1623–1626. 10.1126/science.1110589 15947187

[B34] Wojciszke B. Bazinska R. Jaworski M. (1998). On the dominance of moral categories in impression formation. Pers. Soc. Psychol. Bull. 24, 1251–1263. 10.1177/01461672982412001

