# Supplementary material for: Impressive Words: Linguistic Predictors of Public Approval of the U.S. Congress
Source: Front Psychol. 2016 Feb 23;7:240. doi: 10.3389/fpsyg.2016.00240 (PMC4763029; doi:10.3389/fpsyg.2016.00240)
Supplement: Supplementary file 1 [file DataSheet1.pdf]

Supplementary Material

Impressive Words: Linguistic Predictors of Public Approval of the U.S. Congress

Ari Decter-Frain, Jeremy A. Frimer*

*** Correspondence:** Jeremy A. Frimer: j.frimer@uwinnipeg.ca

1 Introduction

In the supplemental materials, we examined two issues. In part I, we examined whether using an alternative method for calculating the density of agentic and communal language in Congressional debates change the nature of the findings? And in part II, we tested whether the relationship between Congressional language and public sentiment depends on the demographic make-up of Congress?

2 Alternative Agency and Communion Language Assessments

Previous researchers (Hart et al., 2011) developed agency and communion dictionaries for LIWC. Using their dictionaries, we re-analyzed the entire Congressional database and obtained a new set of scores on agency and communion for each month. We believe that the new results suggest that our initial conclusions turn out to be robust with respect to measurement strategy.

Perhaps surprisingly, Hart's agency scores did not converge with the coded agency scores reported in the manuscript, $r(206) = .09$, $p = .18$. Why might they diverge? Hart's dictionary contains words that are explicitly about getting ahead (e.g., "confidence", "certification", "dropout"). The method used in the manuscript may be more implicit. The coders likely asked themselves, "would an agentic person sound like this?" For example, all five of our coders rated filler words, like "uhm", "I mean", and "you know", as indicative of a lack of agency. People who fumble their speech likely do not have the confidence to maintain an agentic persona. Thus, the coded agency scores could be an index of a more implicit form of verbalized agency whereas Hart's system may be more explicit. Given the independence of implicit and explicit systems in dual process theories, the orthogonality found here may be less troubling that it may appear.

The coded method of agency measurement negatively predicted public approval, $r(206) = -.16$, $p = .03$, whereas Hart's agency did not correlate with public approval, $r(206) = -.10$, $p = .14$. This could mean that the difference between these two measures of agency does not pose a problem for our existing interpretation of Congress agency. Further, when we entered both linguistic measures of agency into a regression predicting public approval of U.S. Congress, the coded scores uniquely and negatively predicted approval, $\beta = -.15$, $p = .04$, whereas the Hart dictionary scores did not, $\beta = -.09$, $p = .19$. Hence, the agency implicit in Congressional discourse seems to be particularly off-putting to the American public, whereas the explicit form seems more benign.

Hart's communion scores correlated strongly with the prosocial words dictionary, $r(206) = .97$, $p < .001$, but not with our coded communion scores, $r(206) = -.10$, $p = .16$. Moreover, Hart's

communion dictionary predicted public approval, $r(206) = .49$, $p < .001$. We expected this result because we borrowed heavily from Hart's communion dictionary when we originally developed the prosocial words dictionary (in Frimer et al., 2014). We suspect that Hart's communion scores combine "helping" with "belonging" forms of communion. An analysis supports this notion: When prosocial words and Hart communion were entered together as predictors of public approval, Hart communion's unique contribution to public approval becomes negative, $\beta = -1.04$, $p < .001$, whereas prosocial words still uniquely and positively predicted approval, $\beta = 1.56$, $p < .001$. Hence, these results remain consistent with the interpretations we make in the manuscript—the American public prefers a Congress that talks about helping, but not one that talks about belonging.

We retested hypotheses 1 and 2 using Hart dictionary scores (rather than the coded ones). The results are largely similar to those using coded scores (see Supplementary Tables 1 and 2). Because Hart communion shares most of its variability with the prosocial words dictionary, both regressions display results that resemble table 3 from the manuscript. Prosocial words and Hart's communion dictionary both positively predict approval, even when controlling for outside variables. Helping language remains the key linguistic predictor of public approval. Unlike coded agency, Hart's agency has a small but significant negative correlation with public approval. This finding is not particularly pertinent to our research questions. Another difference between the two analyses occurs upon adding control variables to the model (model 3). In both models, the interaction between agency and communion/prosocial becomes just significant when controlling for other variables (see Supplementary figure 1). To describe the nature of the interaction, we recentered agency at 1 SD above and below the mean, re-ran the regression analysis, and plotted public approval at 1 SD above and below the means on communal or prosocial language. When Congress used highly agentic language, prosocial or communal language did not predict approval. However, when Congress' rhetoric was relatively lacking in agency, communal and prosocial language positively predicted public approval. While this diverges from the results reported in the manuscript, we note that interaction effect sizes are consistently small: $|\beta| = .06$ when coded; $|\beta| = .10$ when using Hart's dictionary. Moreover, the interaction between agency and communion was not a primary focus of the present research.

In summary, using Hart's agency and communion dictionaries, we found that "helping" language is robustly the strongest linguistic predictor of public approval of Congress. Agency is generally a weak negative predictor of public approval. These results are generally consistent with the interpretation we offer in the manuscript.

3 Does Congress' Composition Moderate the Predictors of Approval?

We examined whether the linguistic predictors of Congress depend on the demographic make-up of Congress. In particular, we investigated whether the public favors a Congress that exhibits stereotype-consistent behavior. For example, the public may approve of agentic males and communal females (Eagly, 1987), or agentic Republicans and communal Democratic Party members. To examine these questions, we tested whether the usage of certain types of language (agency, communion, prosocial communion) interact with the composition of Congress (gender balance, partisan composition) to predict public approval. To foreshadow the results, we found several interactions, with some of them suggesting that the public favors stereotype confirming behavior but even more of them suggesting the opposite. These mixed results need to be interpreted with caution, however, insofar as gender balance are conflated with time in this data set.

We collected data on the composition of Congress from www.fec.gov. Between 1996-2014, Congress was comprised of 48.9% Democratic Party members on average (SD = 4.1%), and 18.6% female members on average (SD = 2.7%). Supplementary figure 2 shows the trajectories of gender and party composition of Congress over the period of study.

To test for interactions between linguistic categories and demographic variables, we conducted regression analyses, each of which predicted public approval. In each, we entered standardized agency, communion or prosocial words and one demographic category, along with their 2-way and 3-way interaction terms. If interactions were significant, we decomposed them in follow-up analyses.

3.1 Gender × Language Interactions

3.1.1. Gender × agentic and communal language.

We begin by examining whether agentic and communal language interacts with gender to predict pubic approval. Supplementary Table 3 presents the results. Supplementary figure 3 (both panels) display the strong main effect of gender: a small (5%) increase in female representation predicted a large (23%) decrease in public approval. We also found significant interactions between each linguistic category and gender, but no three-way interaction.

To describe the nature of the two-way interactions (between language and gender), we recentered the gender balance variable at 1 SD above (21% female) and below (16% female) the mean, re-ran the regression analysis, and plotted public approval at 1 SD above and below the means on language. The left panel of Supplementary figure 2 shows that Congressional use of agentic language predicted public disapproval but only when Congress was especially dominated by males. And the right panel of Supplementary figure 2 shows that communal language negatively predicted public approval but only when Congress was less dominated by males. Simply put, the public disapproved of gender-stereotyped language displays (agentic males and communal females). These results do not align with an existing literature that suggests that people respond with agitation and disapproval to stereotype disconfirming displays (e.g., Forster, Higgin & Werth, 2004). Caution is due when interpreting these findings because female representation increased monotonically over the time under study, $r(206) = .98$, $p < .001$. Hence, time and female representation are confounded in this analysis.

3.1.2. Gender × agentic and communal language.

Next, we examined whether agentic and the prosocial form of communal language interacts with gender to predict pubic approval. Supplementary Table 4 present the results of these analyses. We reproduced the same interaction between agency and gender as in the previous analysis. A novel finding in this analysis was an interaction between prosocial language and gender, such that prosocial language had a larger effect on public approval when Congress was relatively female-rich. This finding is consistent with the notion that people approve of stereotype-confirming behavior.

3.2. Language × Party Control Interactions

Next, we ran parallel analyses, only using party control (Democratic vs. Republican) instead of gender as the Congressional composition variable.

3.2.1. Party control × agentic and communal language.

Supplementary Table 5 presents the results of an analysis of language × party composition interactions. Aside from a main effect of communion (high communion predicts low approval), no effects reached significance.

3.2.2. Party control × agentic and prosocial language.

Supplementary Table 6 presents the results of an analysis of language × party composition interactions. We found an interaction between party composition and prosocial language, such that prosocial language was an especially strong predictor of public approval when Republicans controlled Congress (see Supplementary figure 3). This finding is again inconsistent with the notion that people approve of stereotype confirming behavior. We also found main effects of prosocial language (higher > lower) and for party composition (Republican > Democrat). The three-way interaction did not reach significance.

4. Supplementary Figures

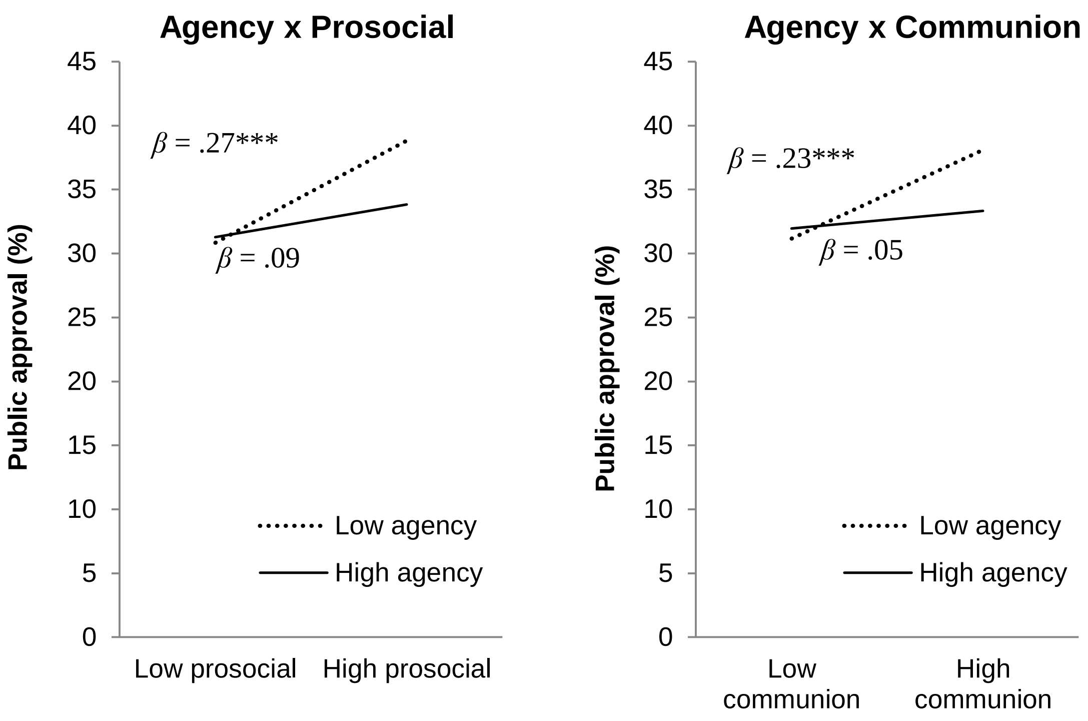

Supplementary Figure 1. The effect of prosocial and communal language on public approval depends on the level of agentic language.

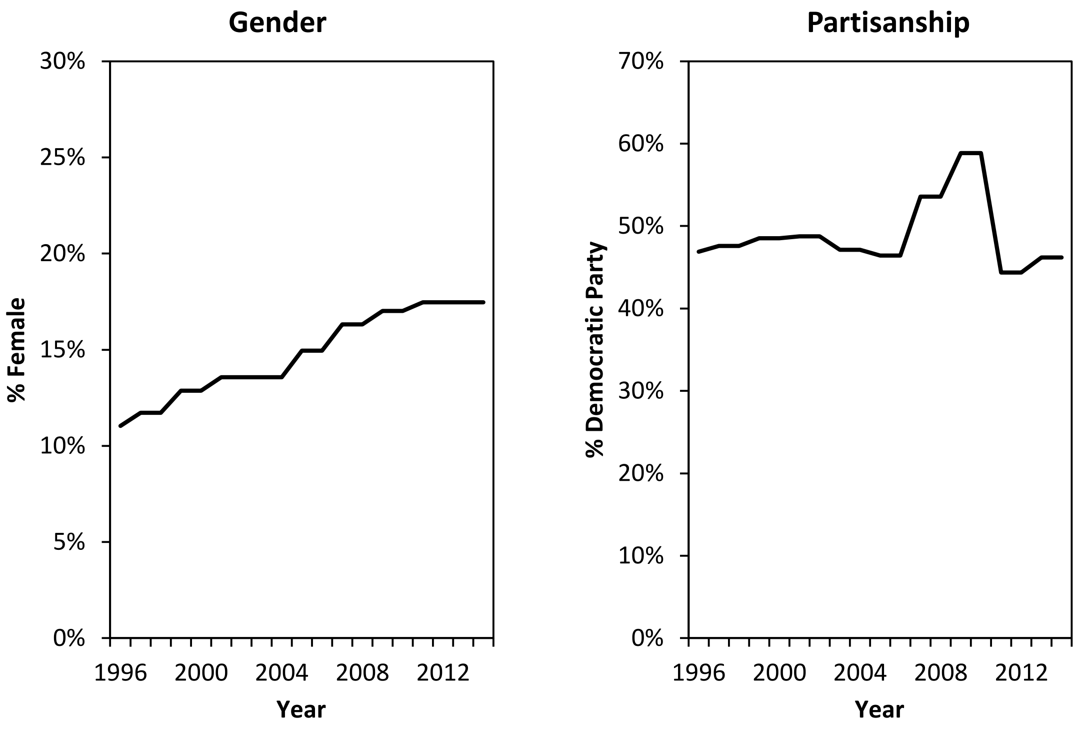

Supplementary Figure 2. Trends in the demographic composition of Congress between 1996-2014.

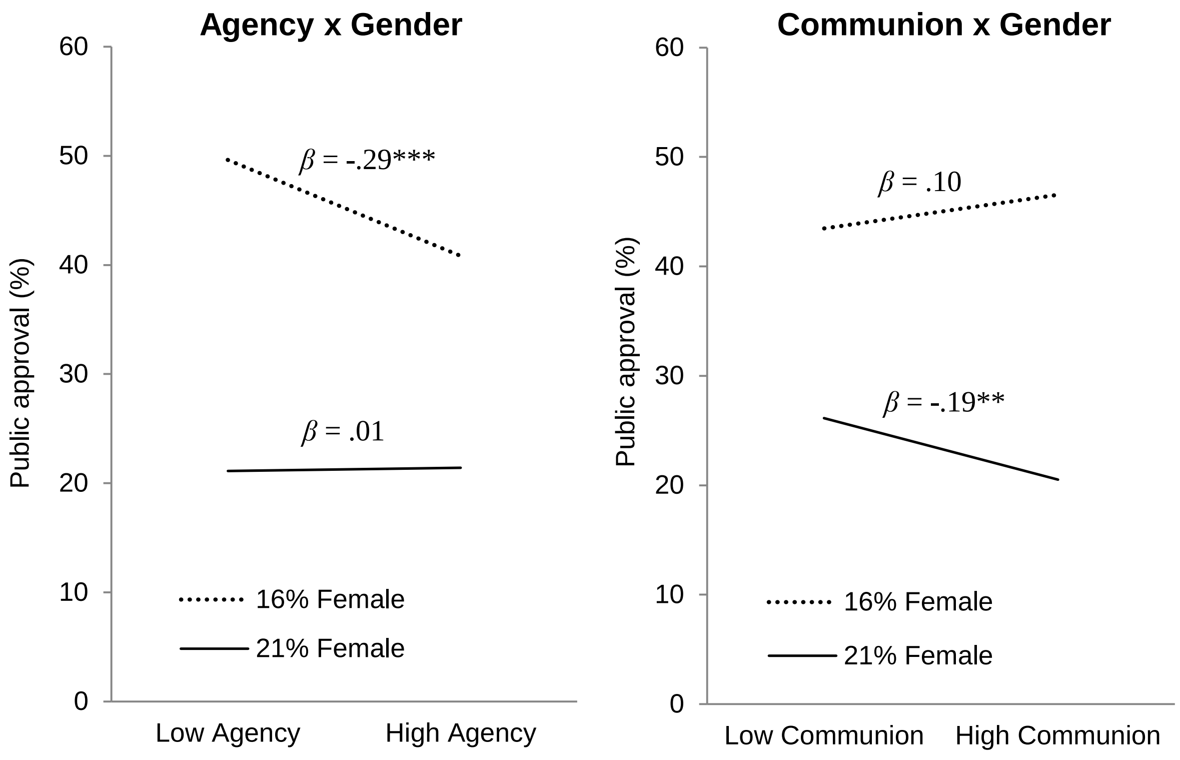

Supplementary Figure 3. The effects of agentic and communal language on public approval depend on the gender balance of US Congress. Each panel shows public approval at +/- 1 *SD* on gender composition (21% vs. 16% female) and at +/- 1 *SD* on language usage. ** $p < .01$, *** $p < .001$

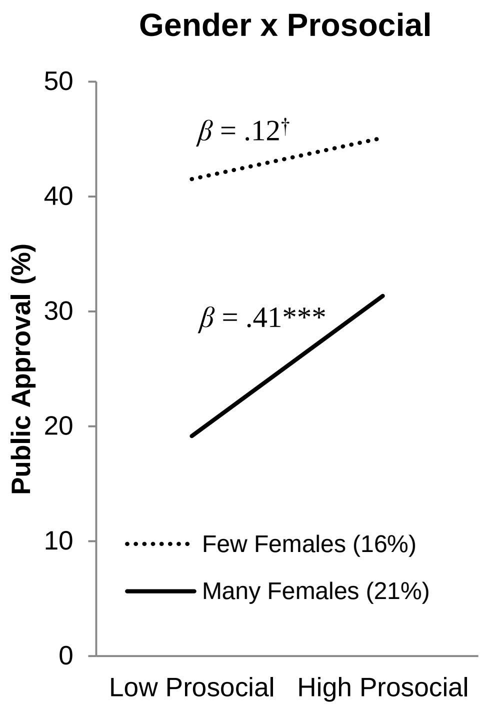

Supplementary Figure 4. The effect of prosocial language on public approval depends on the gender balance of US Congress. † $p < .10$, *** $p < .001$

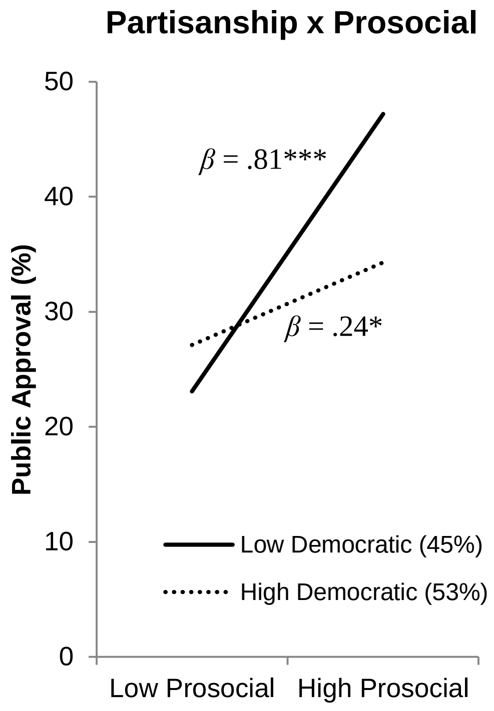

Supplementary Figure 5. The effect of prosocial language on public approval depends on the party composition of the House of Representatives. Prosocial language was an especially strong predictor of public approval when Republicans were in power. * $p < .05$, *** $p < .001$

5 Supplementary Tables

Supplementary Table 1. Results from a test of Hypothesis 1 using agency and communion dictionaries (Hart et al., 2011). Politicians' use of communal language positively predicts their public approval, even when controlling for other variables. * $p < .05$, ** $p < .01$, *** $p < .001$

Predictors	Correlation *r*(206)	Regression Analyses, βs Model 1	Model 2	Model 3
Agency	-.10	-.13*	-.13*	-.07
Communion	+.49***	+.49**	+.49***	+.14**
Interaction			+.02	-.10*
Control Variables				
Exogenous Factors				
World Events				
President's Agency				-.11*
President's Communion				-.04
Unemployment				-.09
Economic expectations				+.06
Endogenous Factors				
Partisan Conflict in Congress				-.11*
Congressional Efficacy				-.05
President vetoes				-.12**
Congressional Composition				
Party				+.16**
Gender				-.60***
R^2 change		.25***	.00	.42***

Supplementary Table 2. Results from a test of Hypothesis 2 using agency and communion dictionaries. Politicians' use of prosocial language positively predicts their public approval, even when controlling for other variables. † $p < .10$, * $p < .05$, *** $p < .01$, *** $p < .001$

	**Correlation**	**Regression Analyses, βs**		
**Predictors**	***r*(206)**	**Model 1**	**Model 2**	**Model 3**
Agency	-.10	-.16**	-.16**	-.07†
Prosocial	+.55***	+.57***	+.57***	+.18***
Interaction			+.08	-.10*
Control Variables				
Exogenous Factors				
World Events				
President Agency				-.12**
President's Prosocial				+.00
Unemployment				-.08
Economic expectations				+.04
Endogenous Factors				
Partisan Conflict in Congress				-.11*
Congressional Efficacy				-.06
President vetoes				-.12*
Congressional Composition				
Party				+.15**
Gender				-.58***
R^2 change		.33***	.00	.34***

Supplementary Table 3. Agentic and communal language independently interacted with the gender balance of Congress to predict public approval. * $p < .05$, ** $p < .01$, *** $p < .001$

	**Correlation**	**Regression Analyses, βs**		
	***r*(206)**	**Model 1**	**Model 2**	**Model 3**
Agency (A)	-.16*	-.17**	-.16**	-.14*
Communion (C)	-.36***	.04	.06	.06
Gender (G; % F)	-.75***	-.77***	-.83***	-.83***
A × C			.01	.00
A × G			.20***	.22***
C × G			-.17***	-.17***
A × C × G				-.04
R^2 change		.59***	.05***	.00

Supplementary Table 4. Agentic and prosocial language independently interacted with the gender balance of Congress to predict public approval.

	**Correlation**	**Regression Analyses, βs**		
	***r*(206)**	**Model 1**	**Model 2**	**Model 3**
Agency (A)	-.16*	-.10*	-.05	-.08
Prosocial Communion (C_p)	.55***	.18**	.24***	.26***
Gender (G; % F)	-.75***	-.66***	-.68***	-.67***
A × C_p			.03	.01
A × G			.21***	.17**
C_p × G			.16**	.18**
A × C_p × G				-.08
R^2 change		.61***	.04***	.00

Supplementary Table 5. Agentic and communal language do not interact with the party composition of Congress to predict public approval.

	Correlation	Regression Analyses, βs		
	***r*(206)**	**Model 1**	**Model 2**	**Model 3**
Agency (A)	-.16*	-.04	-.04	-.04
Communion (C)	-.36***	-.34***	-.34***	-.35***
Party (P; % Dem)	-.07	.01	-.02	.00
A × C			-.03	.02
A × P			.02	-.06
C × P			.03	-.01
A × C × P				.15
R^2 change		.13***	.00	.01

Supplementary Table 6. Prosocial language and party composition of Congress interact to predict public approval of Congress.

	**Correlation**	**Regression Analyses, βs**		
	***r*(206)**	**Model 1**	**Model 2**	**Model 3**
Agency (A)	-.16*	.02	.04	.04
Prosocial Communion (C_p)	.55***	.56***	.55***	.55***
Party (P; % Dem)	-.07	-.06	-.15*	-.15*
A × C_p			.08	.08
A × P			-.03	-.03
C_p × P			-.24***	-.25**
A × C_p × P				.01
R^2 change		.31***	.05**	.00
